# Supplementary material for: DMRforPairs: identifying Differentially Methylated Regions between unique samples using array based methylation profiles
Source: BMC Bioinformatics. 2014 May 15;15:141. doi: 10.1186/1471-2105-15-141 (PMC4046028; doi:10.1186/1471-2105-15-141)
Supplement: Additional file 2 — DMRforPairs output for the comparison of A431-MCF7 and NA17018-NA17105. Please start from the HTML files in each folder. Available via the BMC Bioinformatics website. [file 1471-2105-15-141-S2.zip › 1394847754114233_MOESM2_ESM/A431_MCF7/figures/12634.pdf]

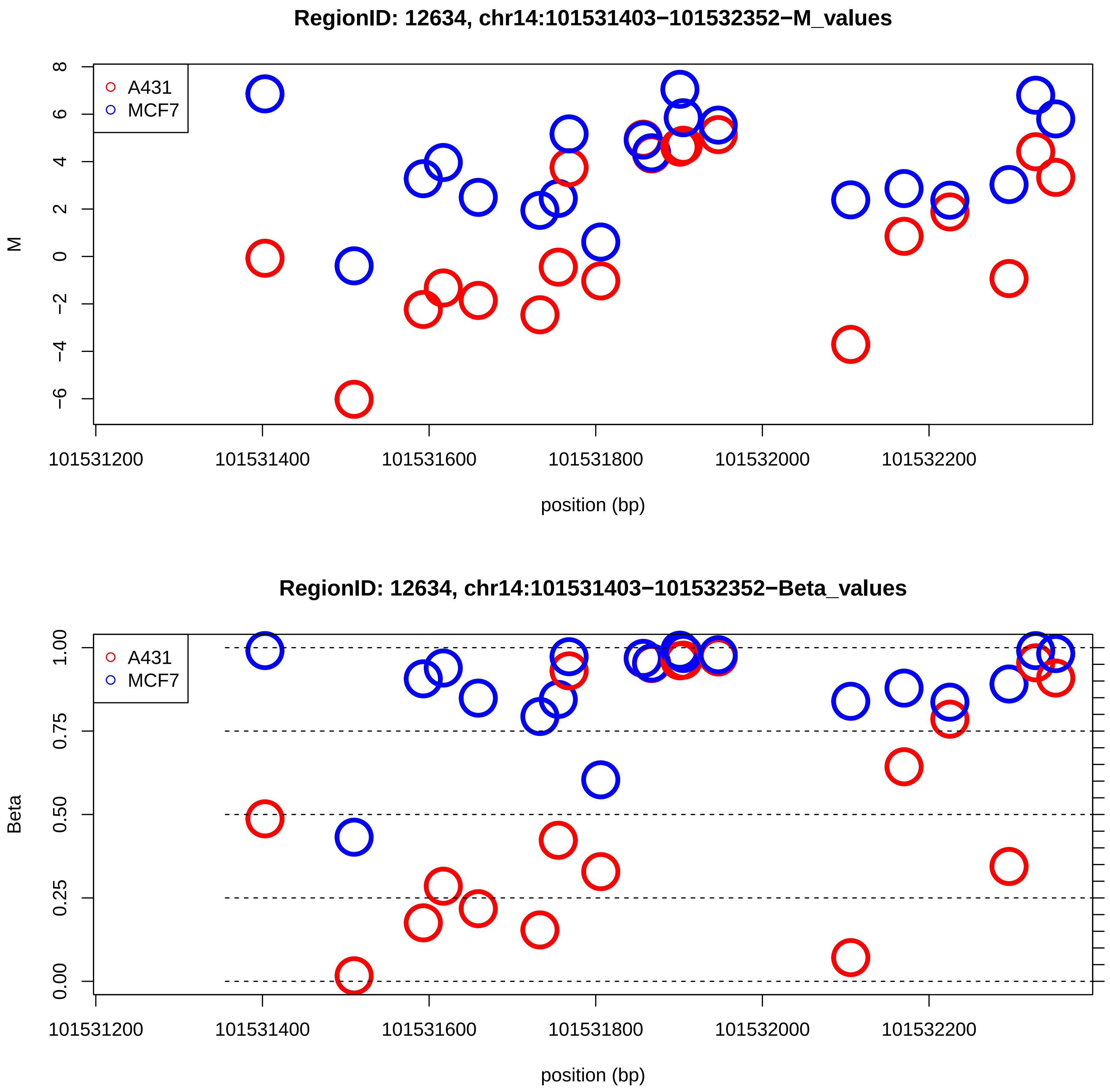
RegionID: 12634, chr14:101531403−101532352−M_values
A431
MCF7
M
8
6
4
2
0
−2
−4
−6
101531200
101531400
101531600
101531800
101532000
101532200
position (bp)
RegionID: 12634, chr14:101531403−101532352−Beta_values
A431
MCF7
Beta
1.00
0.75
0.50
0.25
0.00
101531200
101531400
101531600
101531800
101532000
101532200
position (bp)

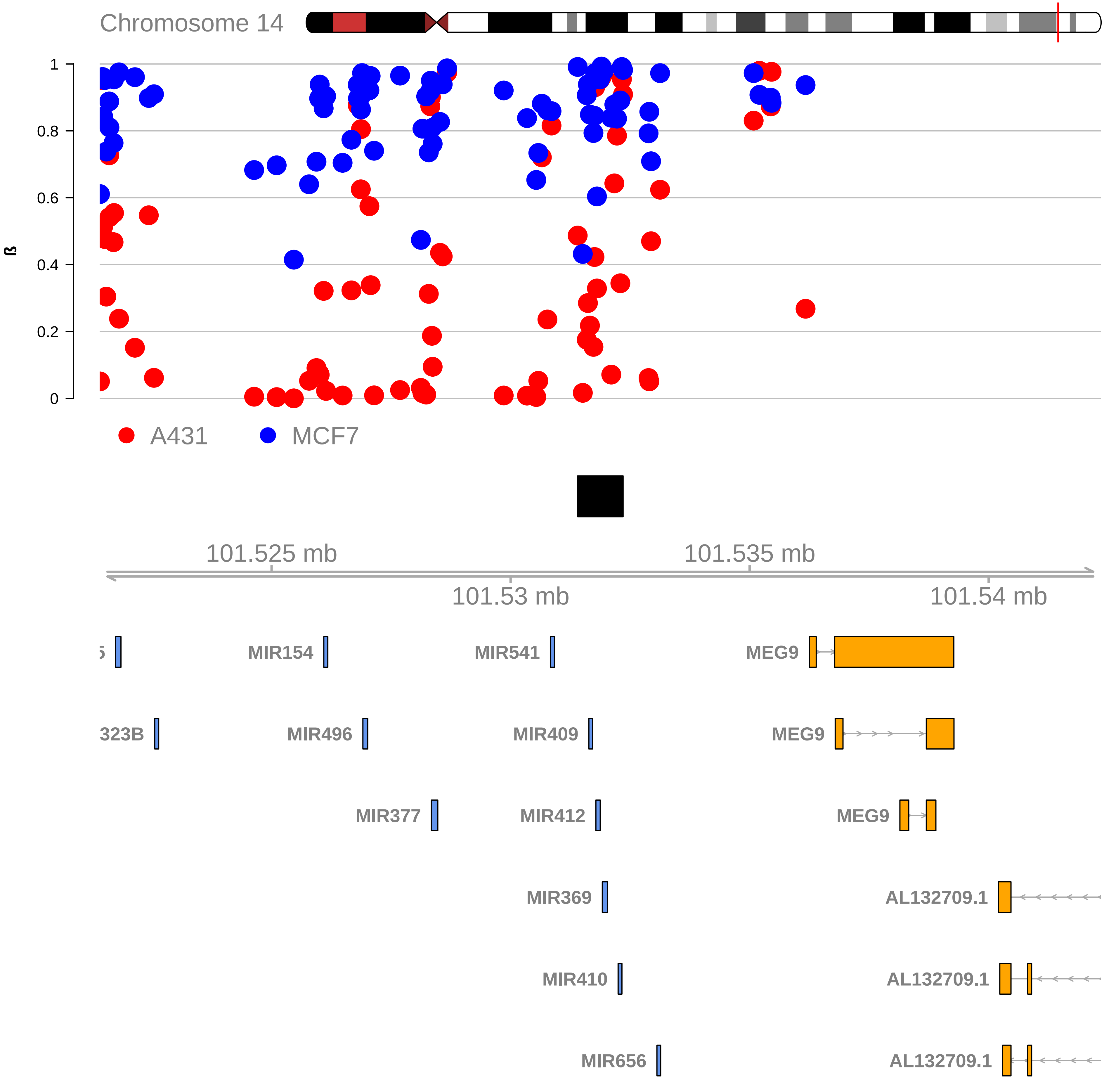

Chromosome 14
1
0.8
0.6
0.4
0.2
0
ß
A431
MCF7
101.525 mb
101.535 mb
101.53 mb
101.54 mb
MIR154
MIR541
MEG9
323B
MIR496
MIR409
MEG9
MIR377
MIR412
MEG9
MIR369
AL132709.1
MIR410
AL132709.1
MIR656
AL132709.1
